# Characterization of the early cellular immune response induced by HPV vaccines

**DOI:** 10.3389/fimmu.2022.863164

**Published:** 2022-07-18

**Authors:** Hella Pasmans, Magdalena A. Berkowska, Annieck M. Diks, Bas de Mooij, Rick J. Groenland, Lia de Rond, M. Alina Nicolaie, Sjoerd H. van der Burg, Jacques J. M. van Dongen, Fiona R. M. van der Klis, Anne-Marie Buisman

**Affiliations:** ^1^ Center for Infectious Disease Control, National Institute for Public Health and the Environment, Bilthoven, Netherlands; ^2^ Department of Immunology, Leiden University Medical Center, Leiden, Netherlands; ^3^ Department of Medical Oncology, Leiden University Medical Center, Leiden, Netherlands

**Keywords:** human papillomavirus (HPV), vaccines, B cells, T cells, innate cells, antibodies

## Abstract

**Introduction:**

Current human papillomavirus (HPV) vaccines consist of virus-like particles (VLPs) which are based on the L1 protein, but they are produced by different expression systems and use different adjuvants. We performed in-depth immunophenotyping of multiple innate and adaptive immune cells after vaccination with bivalent versus nonavalent HPV vaccines.

**Method:**

Twenty pre-menopausal HPV-seronegative women were enrolled and randomized to receive three-doses of either the bivalent or the nonavalent HPV vaccine. Blood samples were collected at multiple time points from baseline up to 7 months after first vaccination. Four extensive EuroFlow flow cytometry antibody panels were used to monitor various immune cell subsets. Additionally, HPV-specific memory B- and T cells were determined by ELISPOT and HPV-specific antibody levels were measured by a VLP-based multiplex immunoassay.

**Results:**

In both cohorts, the numbers of plasma cells expanded in the first week after both primary and tertiary vaccination. HPV16 and HPV18-specific antibody levels and memory B and T-cell responses were higher in the bivalent than in the nonavalent vaccinees one month post third vaccination. For HPV31 and HPV45-specific antibody levels this pattern was reversed. Monocytes showed an expansion one day after vaccination in both cohorts but were significantly higher in the bivalent vaccine cohort. Large heterogeneity in responses of the other cell subsets was observed between donors.

**Conclusion:**

This pilot study showed a consistent response of monocytes and plasma cells after vaccination and a considerable variation in other circulating immune cells in both types of HPV vaccines between donors.

## Introduction

A human papillomavirus (HPV) infection is one of the most common sexually transmitted infections worldwide. Over 200 different subtypes have been identified, including cutaneous types, 15 of these subtypes are classified as oncogenic and an important cause of anogenital and oropharyngeal cancers, but most importantly of cervical cancer. HPV16 and 18 are associated with about 70% of all cervical cancer cases, and 25% are associated with closely related HPV types within the species A9 (HPV16-like: 31,33,35,52,58) and A7 (HPV18-like; 39,45,59,68) (1, 2). Globally, around 800 women die of cervical cancer every day (3).

Currently, there are three licensed, highly efficacious prophylactic vaccines on the market. They provide protection against two (bivalent), four (quadrivalent), or nine (nonavalent) persistent HPV infections, and there is – to some extent- cross-protection against phylogenetically related non-vaccine types. All vaccines comprise virus-like particles (VLPs) which are based on the major HPV capsid protein L1, although they differ by dose. However, the antigens of the vaccines are produced in different expression systems; a baculovirus expression system is used for the bivalent vaccine and a yeast expression system for the quadrivalent and nonavalent vaccines. In addition, different types of adjuvants are used in the vaccines. Whereas the quadrivalent and nonavalent vaccines only use aluminum salts as adjuvant, the bivalent vaccine is formulated with AS04, containing aluminum hydroxide salts and the TLR4 agonist MPL (3-O-deascyl-4’-monophosphoryl lipid A) (4).

Several studies have investigated the differences in immunological responses to these different vaccines, especially comparing responses to the bivalent and the quadrivalent vaccine (5–9). The majority of this work has focused on antibody responses, as they are thought to be the key mediators of protection against infection (10). However, since antibody concentrations wane and memory immunity are being established by the formation and persistence of specific memory B and T cells, cell mediated immunity to HPV antigens is also of importance in the defense against HPV infection. Cellular immunity involves both innate cells acting in the early defense and antigen presentation (11), and adaptive responses in which T helper cells help the generation and (re)activation of B cells, thereby achieving high antibody levels. All three vaccines have shown to induce robust antibody responses against the various vaccine types (9, 12, 13), but the bivalent vaccine induced higher levels of HPV16/18-specific serum antibodies (6, 14) and more robust B-cell responses (7), mostly due to the AS04 adjuvant which is thought to induce a better Th1 response (15, 16).

To the best of our knowledge, innate cellular responses after HPV vaccination have not been studied so far and longitudinal data surrounding the kinetics of immune responses following HPV vaccination is lacking. Insight in the innate responses in relation to the adaptive responses over time would aid the interpretation of the different immune mechanisms of the different HPV vaccines.

The aim of this exploratory study was to increase the knowledge of (early) cellular immune responses after vaccination with the bivalent or the nonavalent HPV vaccine, and to study their possible involvement in HPV-specific memory formation. Therefore, we investigated the kinetics of circulating innate and adaptive immune cell subsets longitudinally by in-depth phenotyping of an extensive set of cell subsets after the first and the third HPV vaccination of young women. Longitudinal kinetics of circulating cells were related to the induction of HPV type-specific antibody and memory B-cell and T-cell responses after completing the vaccination scheme.

## Methods

### Study population and procedures

Participants were recruited among pre-menopausal female personnel of the Dutch National Institute for Public Health and the Environment (RIVM). Potential participants were invited to a pre-study visit and asked to donate a finger prick blood sample to measure the presence of anti-HPV antibodies. During that visit they had the opportunity to ask questions about the study. HPV-seronegative (HPV16/18/31/45) women who were willing to participate were invited for further study consultation and signing the informed consent form according to the Declaration of Helsinki. The study was approved by the Medical Ethical Committees United, Nieuwegein, the Netherlands (study number: NL69015.100.19). Inclusion and exclusion criteria are listed in **Supplementary Table 1**.

**Figure 1:**
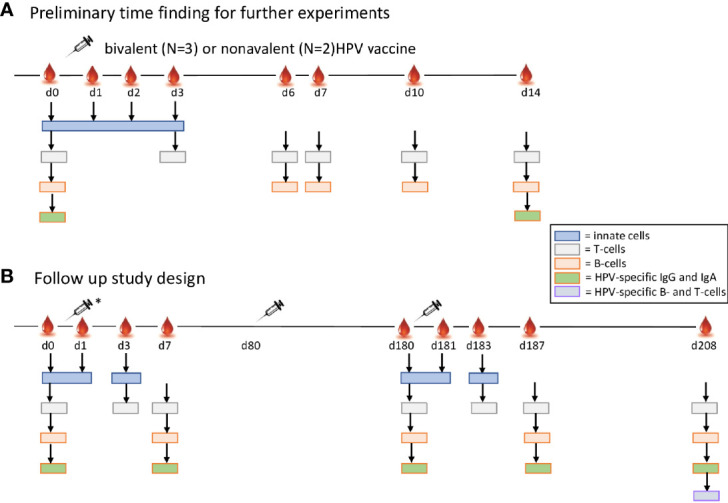
Study design. Adult healthy pre-menopausal women (n=20) seronegative for HPV16/18/31/45 received either the bivalent or the nonavalent HPV vaccine according to a three-dose schedule, the second vaccination at 1 month and the third vaccination at 6 months. **(A)** In the preliminary experiments a blood sample was taken just before vaccination (day 0), and after the first vaccination at day 1, 2, 3, 6, 7, 10 and 14. Numbers of subsets of innate cells (blue), T cells (green), and B cells (red) were studied in 3 bivalent and 2 nonvalent recipients. The arrows indicate the specific time points used per cell type. **(B)** In the follow up (*) blood samples were drawn of the other 7 bivalent and 8 nonavalent vaccine recipients just before vaccination (day 0), and after the first vaccination at day 1, 3, and 7. At two months a second vaccination was given to all 20 study participants (N=10 per cohort). After 6 months (day 180) the third vaccination was given and blood samples were drawn at day 181 (1 day), 183 (3 days), 187 (7 days) and 208 (28 days post third vaccination. At day 0, 7, 180, 187 and 208 HPV IgG serum antibodies specific for HPV16/18/31 and 45 were determined. At 28 days post third vaccination (day 208) PBMCs were isolated for studying HPV-type specific memory B cells and T-cell responses.

Twenty women were enrolled and randomized in a 1:1 ratio to receive either the bivalent (GSK, Rixensart, Belgium) or the nonavalent (Merck, Sharp &Dohme, Kenilworth, NJ) HPV vaccine according to a three-dose schedule (0, 2 and 6 months). Composition and mode of action of the vaccine is described by Schiller et al.^4.^ Please note that in general the bivalent protocol is 0, 1 and 6 months but for comparability we decided to let both cohorts follow the same protocol. Whole blood and serum samples were collected at baseline (day 0) and at pre-defined time points following vaccination. Following primary vaccination, an initial cohort of five volunteers donated blood at eight visits (day 0 (baseline), 1, 2, 3, 6, 7, 10, and 14) to determine the most optimal sampling scheme (**Figure 1A**) and the remaining 15 donors at four visits (day 0, 1, 3, and 7). Then, all volunteers received the first and second booster vaccination and donated samples at day 80, 180 (booster baseline), 181, 183, 187 and 208 (**Figure 1B**).

### Immunophenotyping by flow cytometry

PB-EDTA samples were processed within 2 hours from blood collection. Four different EuroFlow flow cytometry antibody panels were used to monitor kinetics of various immune cell subsets in peripheral blood; a dendritic cell (DC)-monocyte tube (MDC; Van der Pan *et al.*, manuscript under review), a CD4 T cell tube (CD4T) (17), a CD8 cytotoxic T-cell tube (CYTOX tube) and a B-cell and plasma cell tube (BIGH) (18, 19). Additionally, Perfect Count microspheres™ (Cytognos) were used according to the EuroFlow protocol (www.EuroFlow.org) for precise enumeration of cell numbers using the Perfect Count tube (PCT). The CD4 T-cell, CYTOX and the PCT were directly stained on whole blood, using either 100μL (T cells) or 50μL (PCT) of peripheral blood. For the CD4 T cell and CYTOX tube, 100μL PB was stained for 30min at RT in the dark with the corresponding antibody panels. After washing, 100 μL of Reagent A (Fix & Perm, Nordic MUbio, Susteren, the Netherlands) was added and incubated for 15 min at RT in the dark. Cells were washed and 100μL of Reagent B (Fix&Perm™ Nordic MUbio, Susteren, the Netherlands) and intracellular antibodies (Granzyme B, CD154) were added and incubated for 15 min at RT in the dark. After washing, cells were resuspended in 200μL PBS and acquired immediately or were stored at 4°C (max.1 hour) and measured on LSR-Fortessa or Fortessa X20 flow cytometers (BD Biosciences) (stain-lyse-wash protocol, followed by intracellular staining; protocols available at (www.EuroFlow.org). The samples for BIGH and MDC tubes were processed according to the bulk lysis protocol (available at www.EuroFlow.org). Briefly, NH_4_Cl was added to 1.5mL (WBC > 8x10^6^/mL) or 2.0 mL (WBC <8x10^6^/mL) of PB to a final volume of 50mL, and was incubated for 15min on the roller bank at RT. Samples were centrifuged for 10 min at 800g, and washed twice in PBS/0.2%BSA/2mMEDTA/0.09%NaAz. Then, 10x10^6^ cells were stained for 30 min in the dark at RT with corresponding antibody panels (NB: the MDC tube was incubated rolling in the dark). The BIGH staining was followed with the Fix & Perm procedure for intracellular staining with Ig subclasses only. For the MDC tube, which did not require intracellular staining, 2mL of BD lyse (BD FACS™ Lysing solution, BD biosciences) was added, incubated for 10min in dark at RT, and washed. Then, cells were resuspended in 500μL of PBS and immediately measured on one of the BD Fortessa flow cytometers or after storage at 4°C for max 1h.

Daily QC was performed on the flow cytometers according to the EuroFlow guidelines. In short, the photomultiplier tube (PMT) voltages of the flow cytometer were set using BD™ Cytometer Setup and Tracking (CS&T) beads (BD Biosciences) and SPHERO™ Rainbow calibration particles (Cytognos), as previously described (20, 21). For data analysis, Infinicyt software v 2.0 (Cytognos, Salamanca, Spain) was used.

The EuroFlow antibody panels of the different tubes were designed for the PERISCOPE consortium and are elaborately described elsewhere, for the MDC tube (van der Pan *et al.*, manuscript under review), for CD4 (17), the CYTOX and the B cells tube (Patent filed by van Dongen et al. Means and Methods for Multiparameter Cytometry-Based Leukocyte Subsetting. PCT/NL2020/050688, priority date 05 November 2019.) (18). Both absolute cell numbers (cells/μL blood) and ratios over baseline were used through the manuscript. Ratios at day 1, 3 and 7 were calculated over the pre-vaccination baseline (day 0) and ratios at day 181, 183, 187 and 208 were calculated over the third vaccination (pre-second booster) baseline (day 180).

### Detection of memory B cells by ELISPOT

From 18ml of blood collected one month post third vaccination, peripheral blood mononuclear cells (PBMCs) were immediately isolated and stored at -135°CC until analysis. After thawing, B cells were purified from PBMCs by a CD19^+^ selection kit (StemCell Technologies, Vancouver, Canada) and stimulated polyclonally with CPG and cytokines (IL2, IL10 and IL15 10ng/mL) for five days as described previously (22, 23). HPV16, HPV18, HPV31 and HPV45-specific ELISPOT-assays were performed by coating multiscreen-IP plates (Millipore, Burlington, MA) with PBS containing 20 μg/mL HPV16, 18, 31 or 45 VLPs. A suspension of 1x10^5^ B cells was added per antigen in triplicate per participant. Tetanus toxoid (7 flocculation units/mL in PBS) and PBS-coated wells were included as positive- and negative controls, respectively.

For detection of antibody-producing cells as spots, alkaline-phosphatase conjugated goat anti-human IgG was added in combination with BCIP/NBT substrate (Sigma Aldrich, Saint Louis, MI). Spots were analyzed using an Immunospot reader and software (CTL Immunospot S6 Ultra-V Analyzer, Bonn, Germany). Geometric mean (GM) of spot numbers in the PBS-coated wells per participant were subtracted from all HPV-type-specific spot numbers per participant. GM numbers of the corrected HPV-type-specific memory B cells were expressed per 10^5^ B cells. When no HPV-specific spots were detected in any of the wells, values were B cells and set to a value of 0.1 spot/10^5^ B cells.

### Detection of IFN-γ producing cells by ELISPOT

Numbers of HPV-specific IFN-ү-producing cells were measured by ELISPOT. PBMCs were stimulated with VLPs; 4 µg/mL (HPV16, HPV31 and HPV45) and 2 µg/mL (HPV18), in triplicate, in 3x10^5^ cells/well in AIMV medium (Gibco, Waltham, MA) containing 2% human AB-serum (Harlan, Indianapolis, IN), for 4 days at 37°C and 5% CO_2_. Unstimulated and lectin-stimulated cells served as negative and positive controls, respectively. Subsequently, the number of IFN-ү-producing cells specific for HPV16, HPV18, HPV31 and HPV45 was measured using ELISPOT-assays as described previously (24, 25). Spot numbers were counted using an Immunospot reader (version V3.0) and software (version V6.1) (CTL Immunospot S6 Ultra-V Analyzer, Bonn, Germany). Geometric mean (GM) spot numbers of the triplicate wells of unstimulated cells per participant were subtracted from the GM spot numbers of the triplicate wells of HPV-type-specific spot numbers per participant. Subsequently, GM numbers of the corrected HPV-type-specific IFN-ү producing cells were expressed per 1x10^5^ PBMCs. When no spots were detected the value < 1 spot per 3*10^5^ cells was set at 0.5 spot/10^5^ PBMCs.

### HPV-specific antibody levels

HPV16/18/31 and 45 specific IgG and IgA antibody levels in serum at day 7, 14, 180, 187 and in plasma at day 208 after the first vaccination were determined by using a VLP-based multiplex-immunoassay. All VLPs used in this study were provided at cost part by GSK. The VLP-based multiplex has been described elsewhere in detail (26). In short, sera were incubated with HPV-specific VLP-conjugated beads (Bio-Rad Laboratories, Hercules, CA). HPV-specific IgG antibodies were detected using R-phycoerythrin (PE) conjugated goat anti-human IgG (Jackson Immunoresearch, West Grove, PA). The ‘in-house’ control sera and a standard (IVIG Baxter, Utrecht, the Netherlands) were used on each Multiscreen HTS filter plate (Millipore, Burlington, MA). For IgA, a 1/200 dilution of R-PE conjugated goat anti-human IgA (Jackson Immunoresearch, West Grove, PA) was used. HPV-specific antibodies were analyzed using the Bioplex-system 200 with Bioplex-software (Bio-Rad Laboratories, Hercules, CA). IgG antibody levels were expressed in Luminex units (LU) per mL. Semi-quantitative IgA antibody concentrations were expressed in mean fluorescence intensity (MFI).

The presence HPV16, 18, 31 and 45 specific IgG subclasses (IgG1, IgG2, IgG3, IgG4), was determined at day 187. Analysis was performed as described above, by using IgG-isotype-specific mouse anti-human R-PE conjugated secondary antibodies used in 1/500 dilution (IgG1), 1/100 (IgG2-4) (SouthernBiotech, Birmingham, AL). Distributions of IgG-subclasses in percentages were calculated using median fluorescent intensity (MFI) of the IgG subclasses separately in relation to the MFI of the sum of all subclasses, which was set at 100%.

### Statistical analysis

Univariate summaries comprise geometric mean concentrations (GMC) with their 95% confidence intervals (CI). Bivariate summaries comprise Spearman correlations and the permuted version of the Spearman test for pairs of immune cells at each time point.

The permuted version of the Wilcoxon-Mann-Whitney non-parametric test was used to test the associations (described as differences in the manuscript) between each immune cell and vaccination type, separately for each type of point. The permuted version of the Wilcoxon sign-rank test was used to test the distribution of measurements over time.

To compare the changes between two time points for each cell type, the ratios over baseline were used. The calculations carried out comprised the fit of linear mixed models, where the outcome was the log-transformed measurement and determinants were time points and vaccination type. The random effects part was specified as a random intercept. When measurements comprised zero’s, the linear mixed model was fit on the original scale of the measurement and differences were reported instead of ratios.

Additionally, the longitudinal measurements of each immune cell were used to calculate the area under the curve of each participant’s trajectory; the resulting measures were used in an association study in relationship to the vaccination type.

The results of the association tests were corrected for multiple testing with the Benjamini-Hochberg method applied separately to each sub‐study (e.g., the tests of association between cells and vaccination type were treated separately per time point). Reported associations are deemed “significant” because they have passed an application of the Benjamini‐Hochberg (BH) method of multiple testing (targeted at avoiding false positive results by fixing an upper bound on the false discovery rate (FDR) at 15%). These p-values are described as p-adjusted-values throughout the manuscript.

Summary statistics were performed by means of Graphpad Prism V7 (GraphPad, San Diego, CA, USA), while the hypothesis tests were performed in RStudio by means of the coin package (RStudio Team (2020). RStudio: Integrated Development for R. RStudio, PBC, Boston, MA URL http://www.rstudio.com/, version 1.3).

## Results

### Study cohort characteristics

A total of twenty pre-menopausal healthy women aged between 23 and 44 years were included in this study. One participant dropped out at day 1 after the first vaccination and was replaced with a new participant. First, five women vaccinated with either the bivalent (n=3) or the nonavalent (n=2) vaccine were asked to donate blood at baseline and at multiple timepoints directly after the first vaccination (**Figure 1A**). The data of these samples were used to select the timepoints most discriminating in numbers of circulating cells for the total study. At these timepoints data from the first participants were included in the final study results. Here, women received three doses of either the bivalent vaccine (n=10) or the nonavalent HPV vaccine (n=10) at 0, 2 and 6 months (**Figure 1B**). The median age of the participants who received the bivalent vaccine (32.7 years, 95%CI: 28.0-37.4) did not significantly differ from those receiving the nonavalent vaccine (31.6 years, 95%CI: 26.4-36.8).

### No baseline differences between the vaccination cohorts

As the baseline immune status may influence the immune response and leucocyte numbers may fluctuate over time, the ratios in cellular composition at both day 0 and day 180 were used as baseline for immediate vaccine effects (day 0 as baseline for primary vaccination and day 180 for the third vaccination). Indeed, while absolute cell numbers at either of the baselines did not differ between both vaccination cohorts (Mann-Whitney p.adjusted.value <0.3 (corrected for multiple testing)), there were several significant differences in the median cell count of the majority of immune cell subsets between day 0 and day 180 (all p.adjusted.values<0.05) (data not shown).

### Optimal early sampling time points determined based on pilot study

Initial experiments in the first five participants were designed to determine the most discriminating post-vaccination timepoints for blood sampling to measure kinetics of both innate and adaptive immune cells. The numbers of innate cells were evaluated at baseline (day 0), day 1, 2 and 3 post vaccination (27). In donors vaccinated with the bivalent vaccine, the number of monocytes increased at day 1 post vaccination in 2 out of 3 donors (**Supplementary Figure 1A**) and, the numbers of dendritic cells (DCs) showed heterogenous increases between the first five participants (**Supplementary Figure 1A**).

Changes in the numbers of T cells and total B cells at the different timepoints were minor and not consistent between donors (**Supplementary Figure 1B**). Plasma cell numbers however increased at day 7 post vaccination in most donors (**Supplementary Figure 1B**), thereby showing a clear expansion of cells (example shown in **Supplementary Figure 2**). Based on the data of these first five individuals, we selected timepoints where most changes in numbers of specific cells were to be expected. To include relevant time points for studying each major cell subset by limiting the number of sampling to a maximum of 10 per participant, we decided to include the timepoints day 0, 1, 3 and 7 [also used by others (28)] after primary vaccination for studying the cellular kinetics in the remaining 15 participants.

### Increased numbers of circulating monocytes in the first days post vaccination

**Figure 2:**
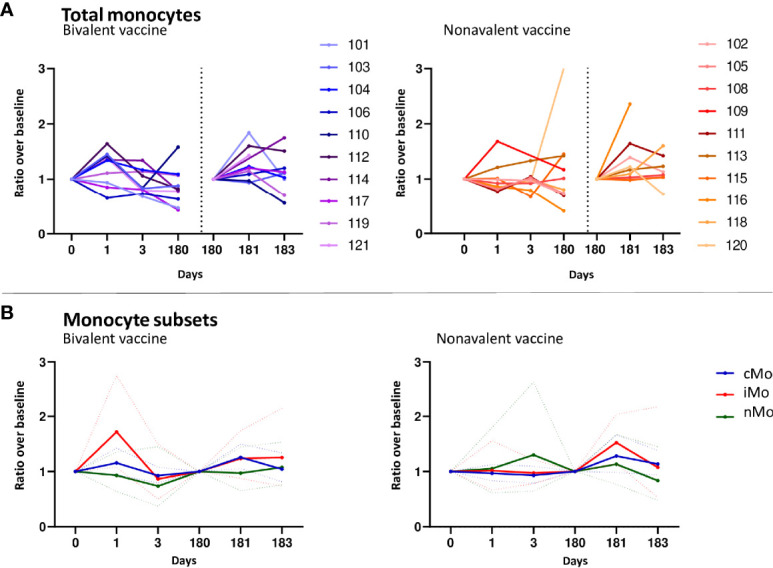
Monocyte responses. **(A)** Kinetics of circulating monocytes upon bivalent (purple-blue) or nonavalent (orange-red) vaccination at day 0, 1, 3, 180, 181 and 183 post vaccination, presented as ratio compared to baseline cell numbers (cells/μL blood) depicted per individual participant. Participant identification by color is indicated on the right. The dotted vertical line split the results post 1^st^ and 3th vaccination. **(B)** Kinetics of monocyte subsets upon bivalent (left) or nonavalent (right) vaccinations at day 0, 1, 3, 180, 181 and 183, presented as the ratio in numbers compared to baseline numbers (cells/μL blood) indicated per vaccine cohort. Solid lines indicate the mean ratio of each cohort and the dotted lines represent the upper and lower confidence intervals. cMo= classical monocytes (blue), iMo= intermediate monocytes (red) and nMo= non-classical monocytes (green). At day 180, participants received the third HPV vaccination, therefore, this day served as a new ‘baseline’ moment to normalize the cell numbers from day 181 and 183, 1 and 3 days post third vaccination, respectively.

The median numbers of monocytes at day 1 compared to baseline, indicated in ratios, showed an increase in the bivalent vaccinated women (1.17, 95%CI 1.003-1.37 (**Figure 2A**), but not in the nonavalent vaccinated women. After the third vaccination, a significant increase in the numbers of monocytes was seen at day 181 in both cohorts, ratio 1.27, 95%CI 1.05-1.55 and 1.28, 95%CI 1.05-1.57, respectively. Next, we investigated changes in monocyte subsets; monocytes can be divided into three phenotypically distinct subsets ranging from the least mature classical (cMo) to intermediate (iMo) to the most mature non-classical monocytes (nMo) (29). These monocyte subsets followed a different pattern over time after each of the vaccines (**Figure 2B**). The bivalent cohort showed a significant early rise in the median ratios of iMo (1.72, 95%CI 1.17-2.53) at day 1 after the first vaccination, but not after the third. In contrast, the median ratio of cMo increased slightly, but significantly at day 181 (1.29, 95%CI 1.08-1.55), but not at day 1 in this cohort. No increases were observed for nMo. In the donors of the nonavalent cohort, the median numbers of iMo and cMos both seemed to increase after the third vaccination, but only the increase in median ratio of cMo was significant (1.26 95%CI 1.04-1.52) (**Figure 2B**). Lastly, statistically significant differences between the two cohorts were observed at day 1 and 3 for the absolute numbers of iMo and cMo (**Table 1**). Overall, a higher number of circulating monocytes was observed shortly after bivalent vaccination than after nonavalent vaccination.

**Table 1:** Cell populations showing significant differences in cell numbers between bivalent and nonavalent vaccine recipients, stratified per timepoint.

Timepoint	Cell population	P-adjusted value
Day 1	Neutrophils	0.002
Day 1	iMo	0.001
Day 1	cMo	0.005
Day 3	Neutrophils	0.021
Day 3	iMo	0.149
Day 3	cMo	0.114
Day 187	HPV16 IgG	0.097
Day 208	HPV16 IgG	0.004
Day 208	HPV16 memory CD4+ T cells	0.071

Only significant differences in numbers of cells between the bivalent and nonavalent cohorts are shown (P.adjusted. <0.15). iMo; intermediate monocytes, cMo; classical monocytes

The total numbers of dendritic cells (DCs) (CD45^+^CD33^+^CD141^+/-^HLA-DR^+^FcERI^+^CD14^-^CD16^-^CD303^+/-^) and mature DCs (mDCs) (CD45+CD33+CD14-dimHLADR+FcERI+CD16+CD141+/-) upon vaccination showed a heterogenous response between donors of both vaccine cohorts (**Supplementary Table 2**).

In the bivalent cohort, ratios of neutrophils increased at day 1 (1.46, 95%CI 1.05-2.02) and 181 (1.42, 95%CI 1.15-1.76) and returned to baseline or lower levels at day 3 and 183 (**Supplementary Figure 3A**). In the nonavalent cohort an increase in numbers of neutrophils was only observed after the third vaccination at day 181 (ratio 1.48, 95%CI 1.18-1.85). Numbers of neutrophils were significantly higher in the bivalent cohort than those in the nonavalent cohort at day 1 and 3 after primary vaccination (**Table 1**).

Basophils are important in allergic response and eosinophils are involved in combating parasites (30), but are not likely to be involved in a response to vaccination. The numbers of basophils and eosinophils fluctuated independent of vaccination, showing variation between donors (**Supplementary Figures 3B, C**, respectively, and **Supplementary Table 2**).

The numbers of Natural killer (NK) cells did not show any consistent change at day 3 or day 7 in neither cohort. However, after the third vaccination there was a clear peak in NK cell numbers at day 183 of the bivalent cohort and at day 187 in the nonavalent cohort (**Supplementary Figure 3D** and **Supplementary Table 2**).

### Subtle changes in T-cell numbers after vaccination, but clear presence of long-term memory T cells

**Figure 3:**
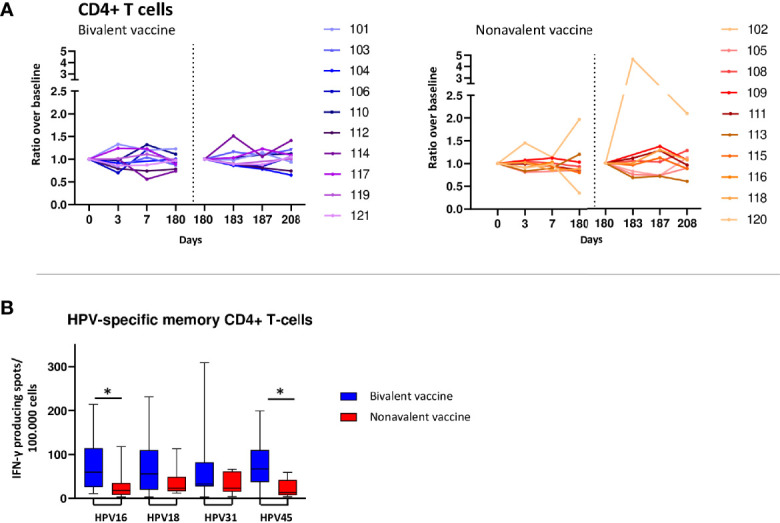
T-cell responses. **(A)** Kinetics of circulating CD4+ T-cells upon bivalent (purple-blue) and nonavalent (orange-red) vaccination at day 0, 3, 7, 180, 183, 187 and 208 post vaccinations presented as ratio in cell numbers compared to baseline cell numbers (cells/μL blood) depicted per participant. Participant identification by color is indicated on the right. At day 180, participants received the third HPV vaccination. therefore, this day served as a new ‘baseline’ moment to normalize the cell numbers from day 181 and 183, 1 and 3 days post third vaccination respectively. The dotted vertical line split the results post 1^st^ and 3th vaccination. **(B)** Numbers of HPV16/18/31/45-type specific memory CD4+ T cells producing IFN-γ at day 208, 28 days post third vaccination. PBMCs have been stimulated with HPV-type specific virus like particles (VLPs) for 4 days after which IFN-y producing cells have been enumerated by ELISPOT assay per 100.000 PBMCs. *indicates significant difference between the cohorts p<0.05.

Although increased numbers of CD4 T cells in the bivalent cohort were observed at day 3 (2 donors) or day 7 (6 donors) post vaccination (**Figure 3A**), no significant changes were observed for median values after primary and third vaccination. In the nonavalent cohort, CD4 T-cell numbers did not show consistent changes after primary and third vaccination. The numbers of CD4 T follicular helper (TFH) cells, which help B cells in the formation of germinal centers, generally followed the kinetics of total CD4 T cells (**Supplementary Table 2**). Overall changes for CD4 T cells and CD4 TFH cell numbers were subtle, but similar between donors.

In general, the numbers of CD4 T-helper 1 (Th1) cells (CD183^+^ CD194^-^ CD196^-^ CCR10^-^) decreased in the first week after primary vaccination in both vaccination cohorts (median ratios 0.69, 95%CI 0.56-0.86 and 0.79, 95%CI 0.64-0.98 at day 3 and 7, respectively in the bivalent cohort and 0.82 95%CI 0.66-1.02 and 0.90 95%CI 0.72-1.11 at day 3 and 7, respectively in the nonavalent cohort). After the third vaccination, the median ratios of CD4 Th1 cells increased to a similar extent in both cohorts at day 3 but the change was only significant in the nonavalent cohort (1.69, 95%CI 1.06-2.71).

The numbers of CD4 Th17 cells (CD183^-^ CD194^+^ CD196^+^ CCR10^-^) showed a significant decrease at day 7 in the bivalent cohort (0.44 95%CI 0.20-0.97), whereas fluctuations in numbers of these cells in the nonavalent cohort after primary vaccination were inconsistent. After the third vaccination, no significant changes were observed in numbers of CD4 Th17 cells in both cohorts.

The median ratios in numbers of CD4 Th22 cells (CD183^-^ CD194^+^ CD196^+^ CCR10^+^) decreased at day 7 in the bivalent cohort (0.38 95%CI 0.15-0.99), but not in the nonavalent cohort. Moreover, after the third vaccination an increase in these ratios was observed in the bivalent cohort, although this was not significant (day 183: 2.06, 95%CI 0.92-4.60 and day 187:2.05, 95%CI 0.89-4.72). Numbers of CD4 Th2 cells (CD183^-^ CD194^+^ CD196^-^ CCR10^-^) in both cohorts had a heterogenous response and showed no significant changes upon vaccination. Likewise, the numbers of CD4 T regulatory (Treg) cells, CD8 naive and memory T cells as well as T cells carrying gamma delta T receptor (TCRγδ) cells fluctuated independently of vaccination and showed a high variation between donors (**Supplementary Table 3**).

Although only subtle changes were observed in total T-cell populations, the long-term HPV16/18/31 and 45-specific IFN-y producing cells were detectable in all donors at day 208, 28 days after the third and last HPV vaccination. The bivalent cohort showed consistently higher numbers of HPV type-specific IFN-y producing cells compared to those of the nonavalent cohort, being significant for HPV16 and HPV45 (p=0.029 and p=0.026, respectively) (**Figure 3B**).

### Plasma cell expansion and maturation 7 days post primary vaccination

**Figure 4:**
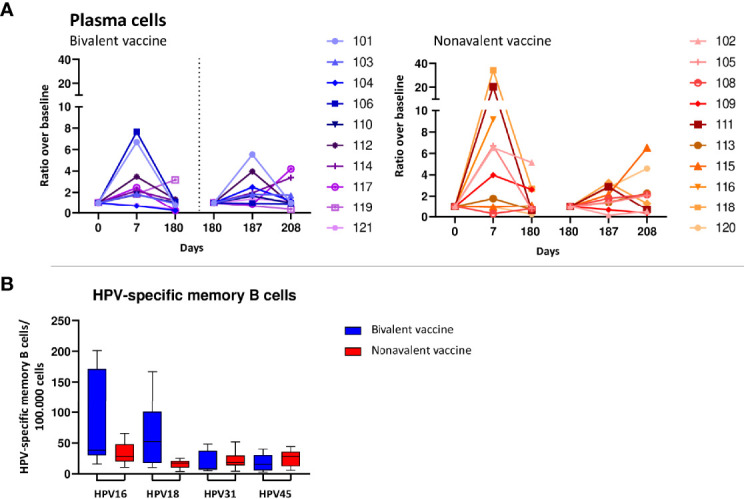
B-cell responses. **(A)** Increases in numbers of plasma cells upon bivalent (purple-blue) and nonavalent (orange-red) vaccination at day 0, 7, 180, 187 and 208 post vaccinations presented as ratio in numbers of cells compared to baseline numbers (cells/μL blood) per participant, presented per vaccine cohort indicated. Participant identification by color is indicated on the right. At day 180, participants received the third HPV vaccination, therefore, this day served as a new ‘baseline’ moment to normalize the cell numbers from day 181 and 183, 1 and 3 days post third vaccination respectively. The dotted vertical line split the results post 1^st^ and 3th vaccination. **(B)** Numbers of HPV16/18/31/45 specific memory B cells at day 208, 28 days post third vaccination. Purified B-cells have been stimulated polyclonally for 5 days and subsequently numbers of HPV-type specific numbers of memory B-cells have been measured by ELISPOT assay in which plates have been coated with HPV-type specific virus like particles (VLPs).

Numbers of total B-cells, naive/preGC and memory B cells did not change in the first week after primary or the third vaccination in both cohorts (**Supplementary Table 2**). However, in both cohorts plasma cells (PC) ratios above baseline showed a strong and consistent increase at day 7 after primary vaccination. This increase was significant in the nonavalent cohort (3.01 95%CI 1.347-6.60) and almost significant in the bivalent cohort (2.15 95%CI 0.98-4.71) (**Figure 4A**). After the third vaccination there was a non-significant trend (0.05<p-value>0.1) towards an increase at day 187 in both cohorts (**Figure 4A**). For the IgG subclasses responses, the increases in cell numbers (expressed as ratio to baseline) as shown in **Supplementary Table 2**. In both the bivalent and nonavalent cohort, a significant increase was seen at day 7 for IgG1 subclasses, (ratio 3.03, 95%CI 1.11-8.31 and 5.80, 95%CI 2.12-15.90 respectively. In the nonavalent cohort a significant increase was also observed for IgG2 and IgM, (ratio 2.27, 95%CI 1.19-4.30 and 5.20, 95%CI 1.53-17.72, respectively. A significant decrease was observed for IgG4 in both the bivalent and nonavalent cohort (0.02, 95%CI -0.02-0.08 and 0.03, 95%CI -0.01-0.06, respectively). After the third vaccination, significant increases were observed in the bivalent cohort at day 187, just for IgG1 (3.11, 95%CI 1.14-8.49) and IgG3 (10.68 95%CI 3.81-29.94). In the nonavalent cohort no significance increase was observed, but for IgG3 a trend to higher numbers of cells was observed (ratio 2.97, 95%CI 1.00-8.82). Furthermore, plasma cells can be divided into maturation stages based on the expression of CD20 and CD138. Most immature plasma cells are CD20^+^CD138^-^, then they become CD20^-^CD138^-^ and most mature plasma cells are CD20^-^CD138^+^. All these maturation stages can be measured in blood (31). Most mature CD20-CD138+ plasma cells showed an increase at day 7 and day 187 (**Supplementary Table 2**).

Although changes in numbers of total memory B cells upon vaccination were not significant within and between cohorts, HPV-specific memory B cells for type 16, 18, 31 and 45 were all detectable at a month post third vaccination (day 208). Interestingly, the bivalent vaccinated cohort showed significantly higher numbers of HPV18-specific memory B cells than those in the nonavalent cohort, p=0.035, and a trend towards higher numbers of HPV16-memory B cells (**Figure 4B**).

### High HPV-specific antibody responses in both vaccination cohorts

**Figure 5:**
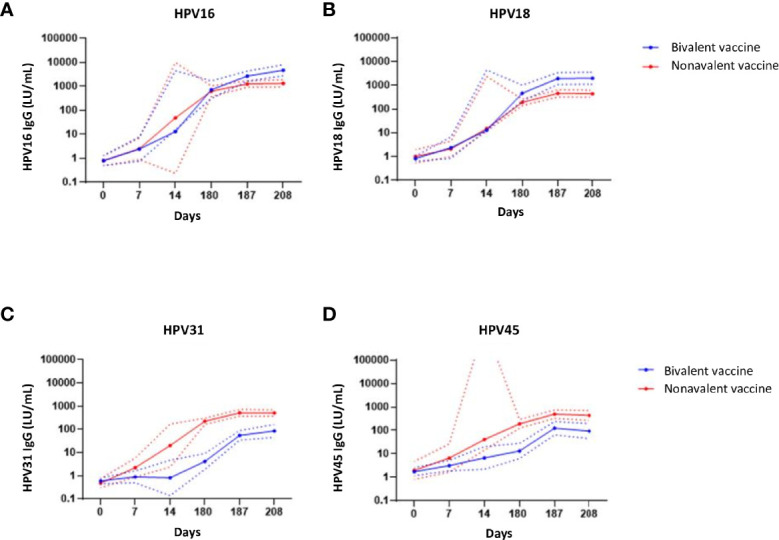
IgG antibody responses. IgG antibodies concentrations (Luminex units/mL blood) specific for **(A)** HPV16**, (B)** HPV-18, **(C)** HPV-31 and **(D)** HPV-45 upon bivalent (blue) and nonavalent (red) vaccinations at day 0, 7, 14, (post first vaccination) and day 180, 187 and 208, respectively 0, 7 and 28 days post third vaccination. The lines represent the geometric mean concentrations (GMCs) over time per vaccine cohort. The dotted lines represent the upper and lower limit of the 95% confidence interval of the geomean. At day 180, participants received the third HPV vaccination. The high upper limit in the nonavalent group for HPV45 is due to the measurement of antibodies at day 14 instead of day 7 in just two donors, which gave a high variation.

HPV-specific IgG antibodies were induced by both vaccines and their levels increased from day 7 up to day 187 after vaccination and seemed to plateau at day 208, 28 days post third vaccination (**Figure 5**). The geomean concentrations for HPV16 and 18-specific IgG levels were significantly higher in the bivalent cohort than those in the nonavalent cohort at day 208, p=0.0011 and p=0.0003, respectively (**Figures 5A, B**). As HPV31 and HPV45 were not present in the bivalent vaccine, HPV31 and -45 IgG-specific antibody levels were significantly higher in the nonavalent cohort compared with those in the bivalent cohort, p<0.0001 and p=0.0015 at day 208, respectively (**Figures 5C, D**). The high upper limit in the nonavalent group for HPV45 is due to the measurement of antibodies at day 14 instead of day 7 in just two donors, which gave a high variation.

The most abundant HPV16,18,31,45-specific IgG subclass at day 187 induced after both the bivalent and nonavalent vaccination was IgG1, followed by IgG3. Very small amounts of IgG2 and IgG4 were found. Nonavalent vaccinated women showed significantly higher HPV16, 18 and 31-specific IgG1 and lower HPV16, 18 and 31-specific IgG3 levels compared to bivalent vaccinated women (**Supplementary Table 3**). HPV-specific IgA levels showed similar patterns as found for IgG but did not show significant differences between the cohorts (**Supplementary Figure 4**).

### Individual kinetics over time per participant in the major cell subsets in both vaccination cohorts

In **Supplementary Figure 5** the absolute numbers of cells for the four major cell populations (monocytes, B cells, plasma cells and T cells) over time are indicated per participant. The graphs are ordered based on the magnitude of the plasma cell expansion. The participants showed a heterogenous response. In seven of them showing a clear plasma cell expansion, no response in their numbers of monocytes was observed. In contrast, in six participants showing a stable or even slight decrease in the numbers of plasma cells a slight increase in the numbers of monocytes was observed. In the remaining three persons showing no response or a decrease in plasma cells, no or a slight decrease in the numbers of monocytes was observed. Numbers of total B cells and CD4 T cells remain similar to baseline values over time, and do not seem to be affected by vaccination. Comparison of the area under the curve per trajectory of number of cells did not result in differences between the vaccination cohorts (data not shown)

### Multidimensional comparisons of kinetics of different cell types

To study the long-term response, we correlated the HPV type-specific IgG levels at day 208, a month post the third vaccination, with numbers of memory B- and T cells, subsets of CD4^+^ T cells, (including Tfh) and plasma cells at various timepoints stratified per vaccine cohort, but no significant correlations were found (all p-values are > 0.05). Likewise, no significant correlations were observed between IgG1-4 serum levels at day 208 and numbers of IgG1-4 plasma cells at day 187 (data not shown).

## Discussion

In this study, we investigated the kinetics of various immune cell subsets in the circulation following primary and third vaccination with the bivalent or nonavalent HPV vaccines using in-depth immunophenotyping by means of state-of-the-art flow cytometry. These kinetics were correlated to HPV-specific antibody levels and numbers of memory B- and T cells post vaccination, and were compared between the bivalent and nonavalent HPV vaccines. The numbers of innate cells, especially monocytes, seemed to expand one day after vaccination in both cohorts and were significantly higher in the bivalent cohort than in the nonavalent cohort. In both cohorts, the numbers of plasma cells expanded significantly in the first week after primary both vaccination and increased after the third vaccination, although not significant. In all other cell subsets, a large heterogeneity in responses between the vaccinees was observed. The HPV16 and 18-specific antibody levels and memory B and T cell responses were higher in the bivalent cohort than those in the nonavalent cohort at one month after the third vaccination. HPV31 and -45 antibody levels, which serotypes are not present in the bivalent vaccine, were elevated in the nonavalent cohort only.

Innate cells, which form the first line of defense against infections, react quickly to vaccine antigens *via* pattern-recognition receptors like toll-like receptors (TLRs). The adjuvant AS04 is described to interact *via* monophosphoryl lipid A (MPL) with TLR4, which is frequently present on antigen presenting cells (APCs) (32, 33). Innate cells, such as monocytes, serve several functions within the immune system, most importantly phagocytosis, antigen presentation and cytokine secretion (34). In this study, the numbers of innate cells have been determined at baseline and day 1 and day 3 after vaccination. Especially the numbers of circulating monocytes, their corresponding subsets, and neutrophils, changed at day 1 post vaccination, although minor as ratios compared to baseline numbers of cells were below 2. Monocytes and dendritic cells react upon vaccination as APCs and migrate to the secondary lymph nodes to be able to present the vaccine antigens, whereas the other innate cells studied have these capacities to a lesser extent (34). Monocytes can be divided into cMo, iMo and nMo (35). In our study we found that absolute numbers of iMo and cMo are higher in the bivalent cohort compared with the nonavalent cohort at the first and third day after vaccination. This can be explained either by the impact the TLR4 agonist present in the adjuvants of the bivalent vaccine by the different pace of immune responses following vaccination or due to daily fluctuations, so we cannot exclude that part of these differences can be attributed to the differences in the kinetics of the immune response. Since changes in the innate compartment are very dynamic and the number of samples is limited, even a minor change in the response time may have a large effect on direct comparisons. A clear peak in iMo numbers in the bivalent cohort at day 1 suggests that a similar expansion of cMo may occur before day 1 and an expansion of nMo between day 1 and day 2, but this is not captured with the current sampling time points. We also observed higher numbers of neutrophils in the bivalent cohort than in the nonavalent cohort within the first days after vaccination, suggesting that the nonavalent vaccine induces a more moderate innate immune response. Induction of Tfh cells play a role *via* the support of activation and differentiation of B cells into Ig-secreting cells (36, 37). However, we found no correlation between numbers of Tfh cells and either plasma cells or antibody levels, presumably by a mismatch in kinetics of circulating Tfh and plasmacells as well as antibody levels at the timepoints included post vaccination (38).

Following the initial wave of the innate cells, the adaptive part of the immune system becomes activated. The numbers of CD4 T cells showed an increase from day 3 up to day 7 after primary vaccination in the bivalent cohort, which was less pronounced in the nonavalent cohort. This is in line with the observed HPV-specific IFN-y responses. After the third vaccination no clear expansion of T cells was seen in both cohorts, similar to what was reported for antigen-specific T cells upon booster vaccination with a hepatitis B vaccine (39) and after Tdap vaccination (40).

Plasma cells, which are responsible for the production of antibodies, showed a clear expansion at day 7 after primary vaccination with any of the two vaccines, which is also clear in the individual plots. This increase was also observed after Tdap vaccination, where plasma cells showed the most prominent increase (40). After the third vaccination, however, this effect was diminished. Since the pilot study, based on which sampling time points were selected, was conducted upon primary vaccination only, it is possible that plasma cell responses upon consecutive antigen-encounter occurred earlier, and therefore were not fully captured by this analysis. In fact, already in the pilot phase, in some donors this response was more prominent at day 6. The role of pre-existing immunological memory was supported by a study showing a plasma cell expansion after the third dose of a rabies vaccination already at day 4 post booster vaccination (41), which was not included in our analysis.

Adjuvants, such as AS04, that activate TLRs are thought to induce antibody class switching (16), that might play a role in the differences in the antibody subclasses found in the two cohorts In the bivalent cohort, an increase in specifically the subclasses IgG1, IgG2, IgG3 and IgA1 was observed. In the nonavalent cohort, IgG1, IgG2 and IgA1 showed the biggest increase. Remarkably IgG3 especially increased after the third vaccination compared with a primary nonavalent vaccination. In contrast, our HPV-specific IgG subclass data show a significantly higher contribution of IgG3 in bivalent-vaccinated women than that in nonavalent-vaccinated women. Spearman correlation analysis did not show any correlation between IgG-subclass-specific plasma cells with HPV-specific IgG-subclass responses at day 187. This could either be explained by kinetics, as the antibodies induced by the plasma cells still need to be formed at day 187. Otherwise, it could be that a part of the induced IgG-subclass-specific plasma cells are not HPV-specific but have been activated by polyclonal stimulation (42). In other studies, bivalent-vaccinated women showed especially an IgG1 and IgG3 antibody profile (43), whereas quadrivalent, containing the same adjuvant as the nonavalent vaccine, showed high levels of IgG4 and IgA in addition to the IgG1 and IgG3 response (44, 45). This, together with our results, suggests that the bivalent vaccine is better capable of inducing an IgG3 response. Since IgG3 is related to a potent pro-inflammatory response (46, 47), this might explain the higher immunogenicity of the bivalent vaccine compared to the nonavalent one.

The higher numbers of HPV16- and 18-specific memory B cells as well as the significantly higher IgG antibody levels for HPV16 and 18 at one month after the third vaccination found in the bivalent cohort compared with the nonavalent cohort, is in line with other studies comparing the bivalent and quadrivalent vaccine (5–7). In individuals that received the bivalent vaccine, cross-reactivity and cross-protection against HPV types absent in the vaccine is observed and is mostly attributed to the AS04 adjuvant (7). Also the HPV-specific CD4 T-cell responses in this study are lower for HPV31/45 in the nonavalent cohort compared to the bivalent cohort showing cross-reactivity in T-cell responses. Although this is observed in many studies (6, 8, 9, 14), the mechanism explaining this is still lacking. Another explanation could lie in the difference in structure of the L1 protein in the two different vaccines since different L1 expression systems are being used for the synthesis of the HPV VLPs. The VLPs used in this study to measure HPV-specific immunity were made with the baculovirus expression system, resembling the VLPs of the bivalent vaccine. However, since others observed similar differences between bivalent- and quadrivalent-vaccinated individuals while making use of recombinant proteins (8), we do not expect that this would lead to any bias. The VLPs of the bivalent L1 proteins produced by using the baculovirus expression vector system contain important conformation-dependent neutralizing epitopes, which closely resemble the native HPV virions that also induce the cross reactivity (48, 49). In contrast, the conformation of the L1 protein VLPs produced in the yeast expression system might be less optimal for inducing cross-protective antibodies.

The immune cellular subsets measured using the EuroFlow tubes are not antigen-specific, which constitute just a little fraction of total memory B and T cells, providing us just information on changes in circulating cell numbers of multiple immune cell subsets upon vaccination. It allows us to monitor innate, B-cell and T-cell immune responses at different time points after vaccination in a highly standardized and reproducible manner. The potent innate immune response upon bivalent vaccination, together with a more optimal antigen presentation might cause the higher memory T-cell responses that subsequently results in a higher plasma cell expansion and the corresponding antibody production.

A limitation of this study was the high heterogeneity observed between donors, which is also described by others (50), together with the low sample size and limited sampling time points, therefore just limited differences between the cohorts were assessed. Moreover, the differences found might be affected by external factors, although all donors were vaccinated, and blood was collected in the same season and time of the day. Therefore, in future studies an extra control cohort receiving a placebo should be considered. It should also be mentioned that in general the bivalent vaccination protocol is 0, 1 and 6 months but for comparability of both cohorts within this study, both cohorts had to follow the same protocol (0, 2, and 6 months). However, we speculate that a time interval of four instead of five months between the second and third vaccination might not strongly affect the cellular kinetics post third vaccination. Moreover, antibody concentrations at 28 days after the third vaccination of the women in this study are comparable to those measured a year post vaccination of teenage girls who had received the second dose at 1 month (23). For this study we only included participants which had no detectable HPV16, 18, 31 and 45 antibodies. However, this does not guarantee that they have not encountered an HPV infection before, as HPV infections can go without having a detectable systemic antibody response and/or infection with other HPV virus types might have occurred. If the study would have been performed in children a clear primary response would be more likely, however this is ethically difficult to obtain permission for. Nevertheless, most HPV infections occur without inducing immunity and the bivalent vaccinated women in this study responded well to the vaccines by producing antibody responses comparable to those observed in teenage girls (23).

Both vaccines induced detectable HPV-specific B and T-cell responses with corresponding high antibody levels, although being higher in bivalent-vaccinated women. Further research in this area could be performed by characterization of HPV-specific B and T cells after vaccination, e.g. by analysis of their antigenic receptor repertoire (51). This could give us insight in the type of memory cells that are formed upon either bivalent or nonavalent vaccination, possibly giving us an explanation of the observed cross-protection in bivalent-vaccinated women.

The HPV field is just beginning to understand the potential implications of innate and/or adaptive immune signatures and adjuvant effects on the generation of effective adaptive immune responses upon vaccination. Further insight into the impact of the cellular response on the efficacy of the currently used vaccines may be of importance. This will increase the knowledge on the linkage of innate immune response to long term immunity.

## Data availability statement

The original contributions presented in the study are included in the article/**Supplementary Material**. Further inquiries can be directed to the corresponding author.

## Ethics statement

The studies involving human participants were reviewed and approved by Medical Ethical Committees United, Nieuwegein, the Netherlands (study number: NL69015.100.19). The patients/participants provided their written informed consent to participate in this study.

## Author contributions

HP, AB, AD, FK, MB, JD, and SB contributed to the design and implementation of the research. AN and HP to the statistical analysis of the results. HP drafted the manuscript and all authors reviewed the manuscript. HP, BM, RG, and LR performed the measurements. All authors contributed to the article and approved the submitted version.

## Conflict of interest

MB, AD, and JD report inventorship of the patent “Means and methods for multiparameter cytometry-based leukocyte subsetting” (NL2844751, PCT/NL2020/050688, priority date 5 November 2019), owned by the EuroFlow Consortium.

The remaining authors declare that the research was conducted in the absence of any commercial or financial relationships that could be construed as a potential conflict of interest.

We thank GlaxoSmithKline Biologicals SA for providing at cost part of the VLP’s that we used in our assays. GlaxoSmithKline Biologicals SA was provided the opportunity to review a preliminary version of this manuscript in order to ensure the protection of its proprietary information and intellectual property, but the authors are solely responsible for final content and interpretation.

## Publisher’s note

All claims expressed in this article are solely those of the authors and do not necessarily represent those of their affiliated organizations, or those of the publisher, the editors and the reviewers. Any product that may be evaluated in this article, or claim that may be made by its manufacturer, is not guaranteed or endorsed by the publisher.
