# Supplementary material for: Characterization of the early cellular immune response induced by HPV vaccines
Source: Front Immunol. 2022 Jul 18;13:863164. doi: 10.3389/fimmu.2022.863164 (PMC9341268; doi:10.3389/fimmu.2022.863164)
Supplement: Supplementary file 1 [file DataSheet_1.docx]

**Supplementary information**

**Supplementary Table 1:** Inclusion and exclusion criteria of the EVI study

| **Inclusion criteria** | **Exclusion criteria** |
| --- | --- |
| - Seronegative for high risk-HPV vaccine types (16,18,31,45); - Female; - Normal general health; - Pre-menopausal; - Willing to receive HPV vaccination; - Provision of written informed consent; - Willing to adhere to the protocol and be available during the study period. | - Present evidence of serious disease(s) within the last 3 months before inclusion requiring immunosuppressive or immune modulating medical treatment, such as systemic corticosteroids, that might interfere with the results of the study; - Chronic infection; - Known or suspected immune deficiency; - History of any neurologic disorder, including epilepsy; - Previous administration of serum products (including immunoglobulins) within 6 months before vaccination and blood sampling; - Known or suspected allergy to any of the vaccine components (by medical history); - Previous vaccination with any HPV vaccine; - Pregnancy; - Participation in another vaccination/ medicine study. |

**Legend Supplementary Table 2 in Excel file**

Absolute numbers of the major populations of B-cells (tab 1), T-cell and NK cells (tab 2) and innate cells (tab 3) measured per timepoint were given as background information. Data are given as geomean cell numbers per µl/blood (first columns) and lower (second) and upper confidence intervals (third column) for both the bivalent cohort (left) and nonavalent cohort (right).

**Supplementary Table 3.** HPV16,18, 31 and 45-specific IgG-subclass expressed as percentages of the total HPV-specific IgG production. For each HPV serotype and IgG-subclass, we show the difference between the bivalent and nonavalent cohort and the corresponding 95% confidence interval. IgG subclass production was measured at day 208 (28 days after the third vaccination).

| **HPV16** | | | | | | | | |
| --- | --- | --- | --- | --- | --- | --- | --- | --- |
|  | **IgG1** | | **IgG2** | | **IgG3** | | **IgG4** | |
|  | bivalent | nonavalent | bivalent | nonavalent | bivalent | nonavalent | bivalent | nonavalent |
| Mean | 53.92* | 74.95* | 0.11* | 0.23* | 45.9* | 24.67* | 0.07* | 0.15* |
| 95% CI | 39.3-68.5 | 67.2-82.8 | 0.04-0.17 | 0.15-0.31 | 31.3-60.5 | 16.9-32.5 | 0.03-0.11 | 0.12-0.18 |
| **HPV18** | | | | | | | | |
|  | **IgG1** |  | **IgG2** |  | **IgG3** |  | **IgG4** |  |
|  | bivalent | nonavalent | bivalent | nonavalent | bivalent | nonavalent | bivalent | nonavalent |
| Mean | 49.7* | 86.9* | 0.15 | 0.17 | 50.0* | 12.8* | 0.13 | 0.16 |
| 95% CI | 35.4-64.1 | 81.2-92.7 | 0.07-0.24 | 0.11-22 | 35.6-64.4 | 7.0-18.5 | 0.05-0.22 | 0.14-0.19 |
| **HPV31** | | | | | | | | |
|  | **IgG1** |  | **IgG2** |  | **IgG3** |  | **IgG4** |  |
|  | bivalent | nonavalent | bivalent | nonavalent | bivalent | nonavalent | bivalent | nonavalent |
| mean | 20.4* | 79.9* | 0.26 | 0.21 | 79.3* | 19.7* | 0.02* | 0.17* |
| 95% CI | 13.6-27.2 | 72.7-87.1 | 0.0-0.62 | 0.13-0.29 | 72.4-86.2 | 12.5-26.6 | 0.0-0.06 | 0.15-0.19 |
| **HPV45** | | | | | | | | |
|  | **IgG1** |  | **IgG2** |  | **IgG3** |  | **IgG4** |  |
|  | bivalent | nonavalent | bivalent | nonavalent | bivalent | nonavalent | bivalent | nonavalent |
| mean | 68.6 | 76.7 | 0.16 | 0.28 | 31.2 | 22.6 | 0.07 | 0.46 |
| 95% CI | 56.4-80.8 | 61.9-91.4 | 0.0-0.34 | 0.0-0.66 | 19.1-43.2 | 8.8-36.4 | 0.0-0.14 | 0.0-1.10 |

*p<0.05 indicates a significant difference between the bivalent and nonavalent cohort for that specific subclass and HPV serotype.


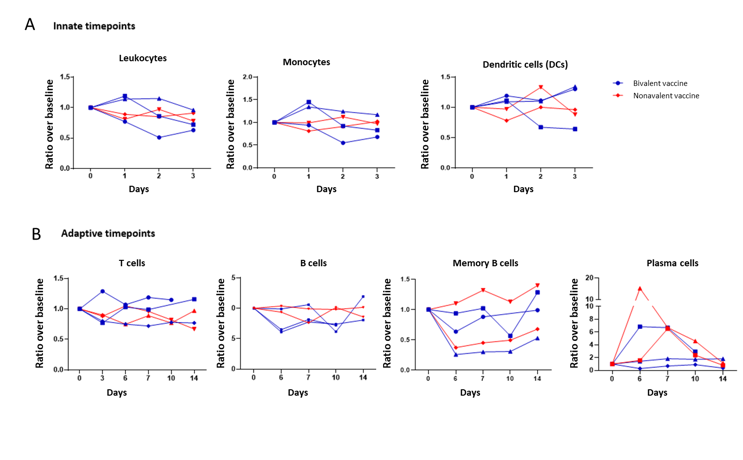


**Supplementary Figure 1. Numbers of circulating cells per mL blood in the preliminary time finding experiment.** Kinetics of A) innate cells: leukocytes, monocytes and dendritic cells (DCs) at day 0, 1, and 3 post vaccinations and B) adaptive cells: T cells, B cells, memory B cells and plasma cells at day 0, 3, 5, 6, 7, 10 and 14 post vaccination. All kinetics are presented as ratio over baseline numbers (cells/μL blood) and each line represents a participant. Ratios of the first three bivalent vaccinees (blue) and two nonavalent vaccinees (red) are shown.


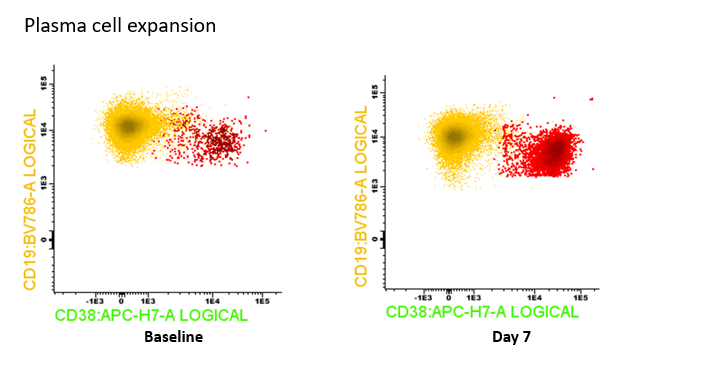


**A B**

**Supplementary Figure 2. Plasma cell expansion post vaccination**. Example of a clear plasma cell expansion from A) baseline to B) day 7 post vaccination of a representative bivalent donor. Plasma cells are depicted in red and have high CD38 expression, whereas memory B cells are depicted in yellow and do not show high CD38 expression.


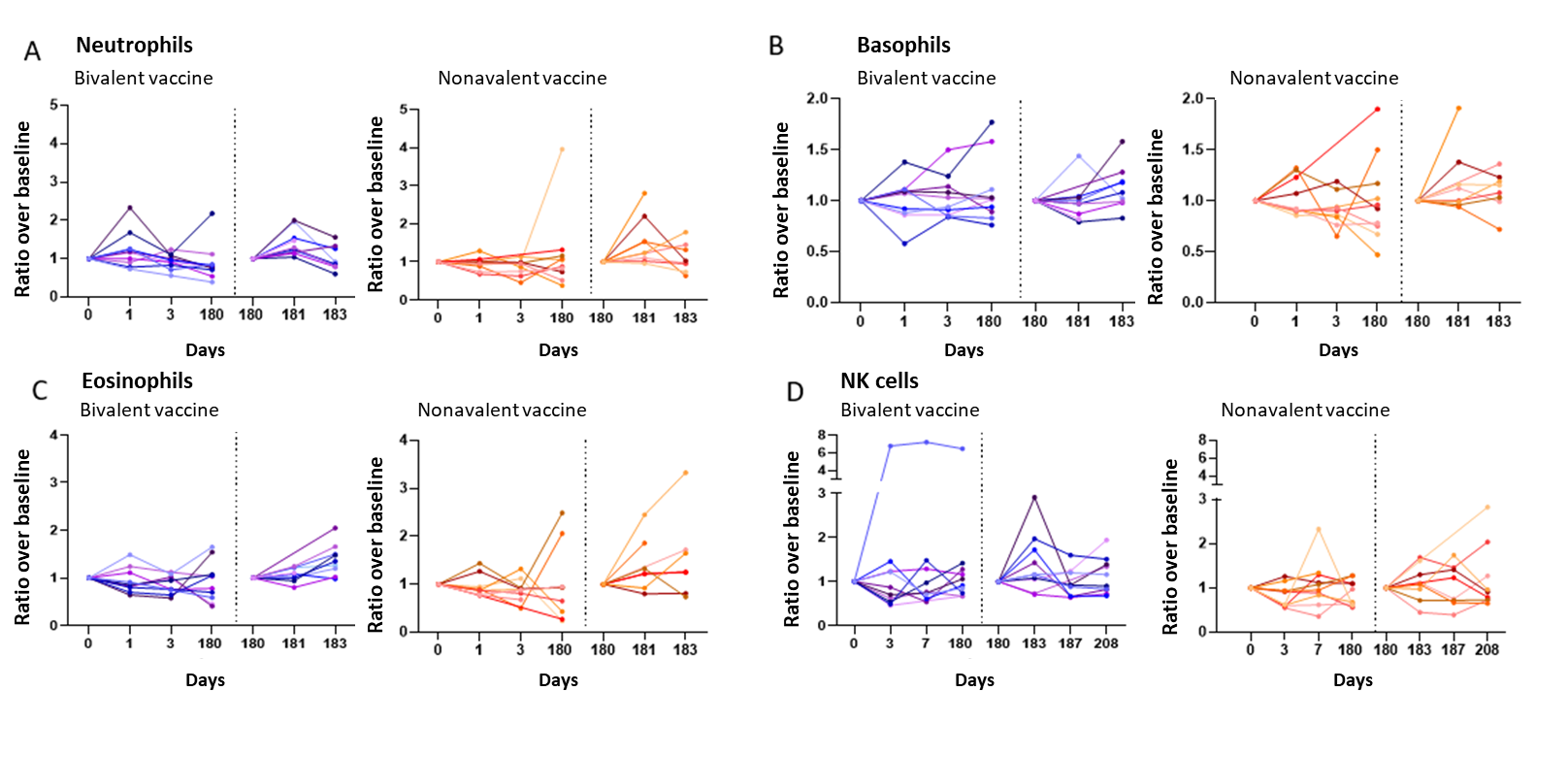


**Supplementary Figure 3. Numbers of granulocytes and natural killer cells.** Kinetics of circulating A) neutrophils B), basophils , C) eosinophils and D) NK cells shown presented as ratio over baseline upon bivalent (purple-blue) and nonavalent (orange-red) vaccination at day 0, 1, 3, 7 180, 181, 183 and 187 post vaccination. Fluctuations of cells are presented as ratio compared to baseline value. The first vaccination is given at day 0, and the third vaccination is given at day 180.

**Supplementary Figure 4. The IgA antibody responses**. Levels of HPV 16/18/31/45 specific serum IgA antibodies upon bivalent (blue) and nonavalent (red) vaccination at day 0, 7, 14, 180, 187 and 208 (28 days post third vaccination). The third vaccination is given at day 180. Semi-quantitative IgA antibody concentrations were expressed in mean fluorescence intensity (MFI). The solid lines indicate the geomean and the dotted lines indicate the upper and lower 95% confidence intervals.


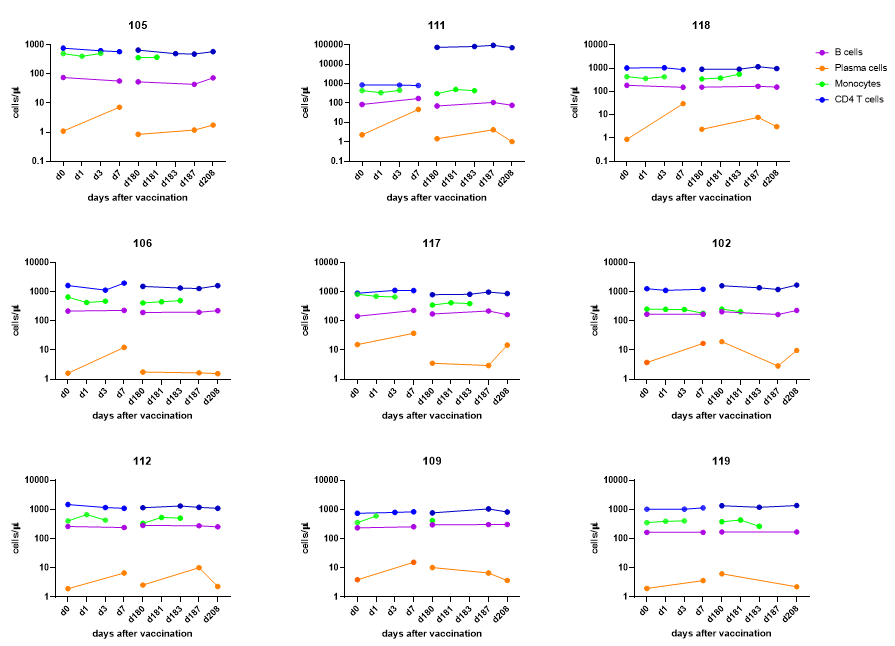


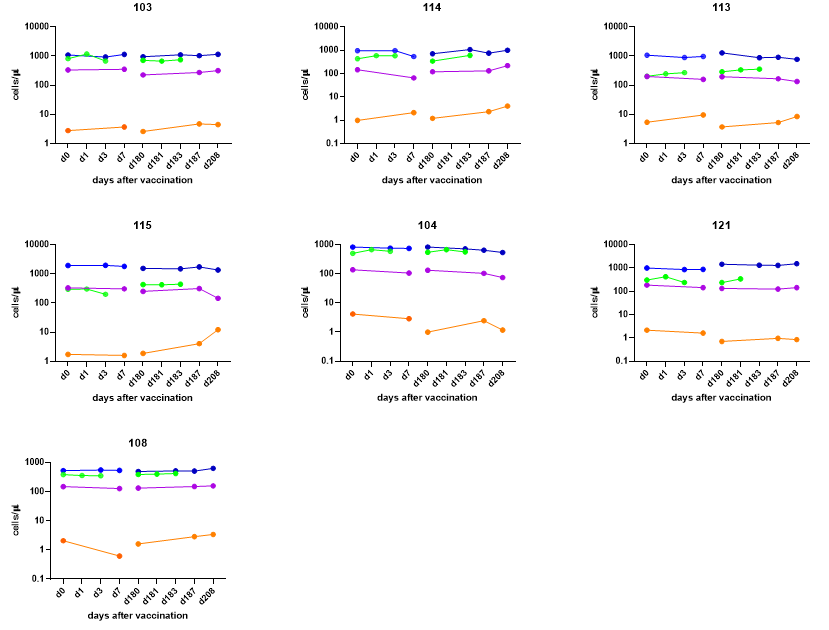


**Supplementary Figure 5. Absolute numbers of cells per donor over time.** The absolute number of cells (cells/μL blood) of four important cell populations: monocytes (green), B-cells (purple), plasma cells (orange/red), and CD4 T cells (blue), at day 0, 1, 3, 7, 180, 183, 187 and 208 time post vaccination per donor. The first vaccination is given at day 0 and the third vaccination is given at day 180.
